# Irritable Bowel Syndrome: Treating the Gut and Brain/Mind at the Same Time

**DOI:** 10.7759/cureus.43404

**Published:** 2023-08-13

**Authors:** Maleesha Jayasinghe, John A Damianos, Omesh Prathiraja, Melysze D Oorloff, Gairu M Nagalmulla K, Adithya Nadella, Dilushini Caldera, Ali Mohtashim

**Affiliations:** 1 Medicine, Nanjing Medical University, Nanjing, CHN; 2 Gastroenterology and Hepatology, Mayo Clinic, Rochester, USA; 3 Medicine and Surgery, Nanjing Medical University, Nanjing, CHN; 4 Internal Medicine, Nanjing Medical University, Nanjing, CHN; 5 Medicine, 37 Military Hospital, Accra, GHA; 6 Medicine, Allama Iqbal Medical College, Lahore, PAK

**Keywords:** diagnosis of ibs, fodmap diet, gut-brain axis, gut-brain connection, irritable bowel syndrome, irritable bowel disease, ibs treatment

## Abstract

Irritable bowel syndrome (IBS) is one of the most common functional gastrointestinal (GI) disorders in the world. Although IBS does not affect a person's life span, it can significantly influence their quality of life. The treatment of IBS should be tailored to each patient's specific symptomatology because it can often be difficult to manage. Given that the pathogenesis of IBS is not well understood, it places a tremendous load on healthcare resources. Over the years, IBS has been described as either a simple GI disorder or a more complex multi-symptomatic gut-brain axis disorder. Many persons with IBS have psychological issues in addition to gastrointestinal symptoms, offering the door to non-pharmacological therapies such as cognitive behavioral therapy, gut-directed hypnosis, or psychodynamic interpersonal therapy. Non-pharmacological therapies with no side effects should be used as first-line therapy. Diet, exercise, microbiota-targeted therapies, and psychological treatments are among the most significant interventions. This review goes into the details of all the non-pharmacological interventions that can be used to treat IBS.

## Introduction and background

**Figure 1:**
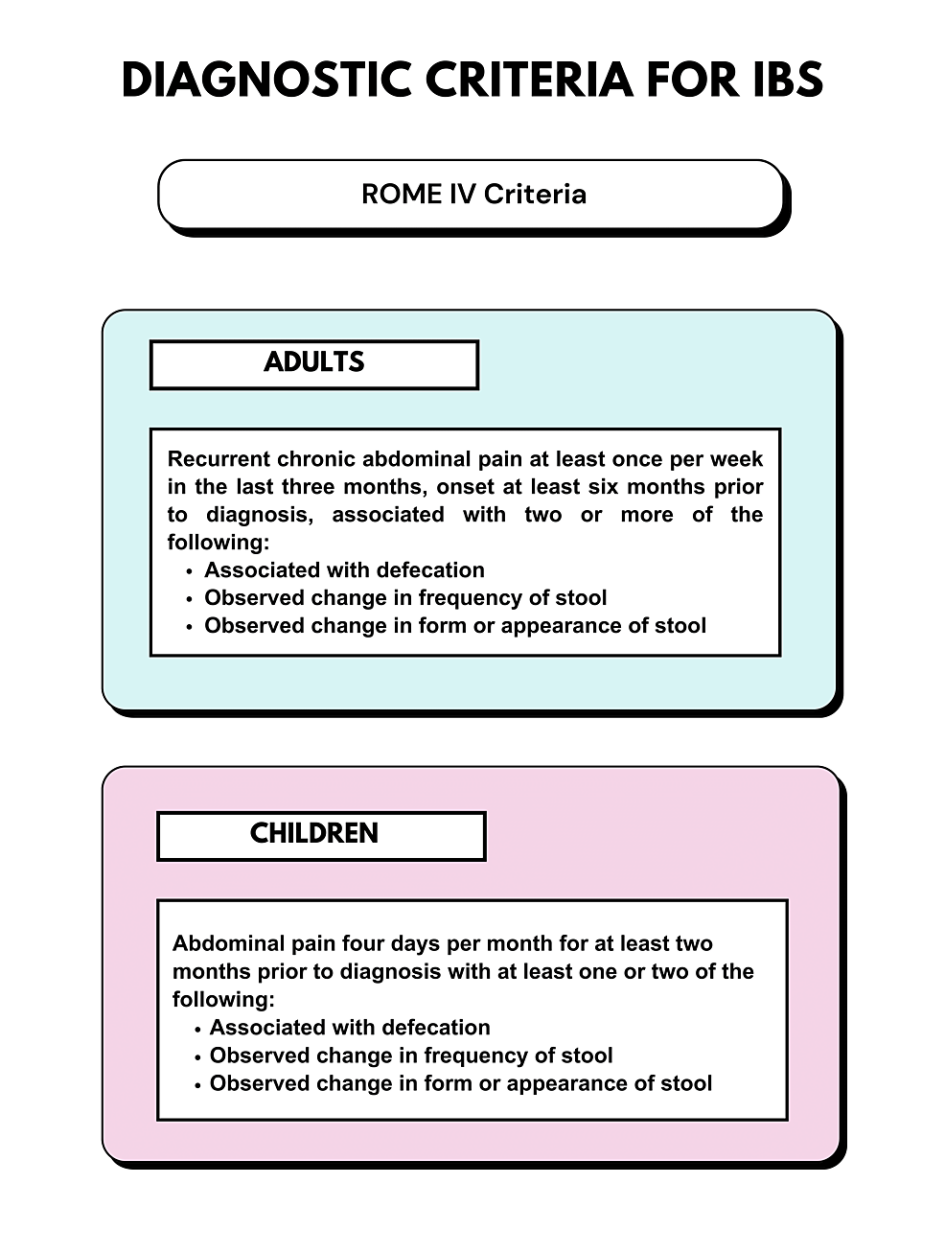
Irritable bowel syndrome (IBS) diagnostic criteria Original figure created by Dr. Adithya Nadella

Irritable bowel syndrome (IBS) is one of the most prevalent functional bowel disorders, with a prevalence of 7%-16% in the United States and 4%-10% worldwide; it is most commonly seen in women and young people [1-3]. According to the 2016 Rome IV criteria, IBS is diagnosed on the basis of recurrent abdominal pain, bloating, and stool irregularity in terms of stool frequency and form, and symptoms can range from mild to debilitating (Figure 1) [4]. The patients should have chronic symptoms that occur at least once per week on average in the previous three months, lasting at least six months [3].

IBS consists of four subtypes: IBS with constipation (IBS-C), IBS with diarrhea (IBS-D), mixed IBS (IBS-M) or alternating IBS (IBS-A), and IBS unsubtyped (IBS-U) [5]. Symptomatic diagnosis of IBS is made along with physical examinations, including digital rectal examination and screening tests such as hemoglobin, C-reactive protein, and celiac serology to rule out organic disease [6]. The etiology of IBS is multifactorial and has not been completely established. Recent studies have proposed several hypotheses that indicate IBS is related to dysfunction in the hypothalamic-pituitary-adrenal axis, neuroendocrine alterations, visceral hypersensitivity, and genetic factors (Table 1) [7].

**Table 1:** Proposed pathophysiological causes of IBS and their proposed methods of pathogenesis IBS: irritable bowel syndrome Original table created by Dr. Adithya Nadella

Authors	Type of study	Proposed pathophysiologic causes of IBS
Ng et al. [7]	Review	There has yet to be a definite and established pattern of an immune response, but increasing evidence does support the inflammation-immunological etiopathogenesis of IBS. Increased mast cell density and activity of the gut may be seen along with visceral hypersensitivity symptoms. Post-infectious IBS and infective gastroenteritis could cause systemic inflammation and altered microbiome diversity leading to chronic low-grade subclinical inflammation. Neuroinflammation is another aspect involved in the pathophysiology via the gut-brain axis, which alters neuroendocrine pathways, glucocorticoid receptor gene, and dysregulated hypothalamic-pituitary-adrenal axis.
Shrestha et al. [8], Hasler et al. [9]	Review	Visceral hypersensitivity is one of the main factors for the pathogenesis of IBS, caused due to the increased perception of luminal stimuli and contributes to abdominal pain. Visceral hypersensitivity is primarily observed in visceral pain pathways at peripheral, spinal, and supraspinal levels, demonstrating increased sensitivity. Mast cells release histamine, proteases, prostaglandins, and cytokines and may increase hypersensitivity and permeability defects. They also disrupt epithelial barrier function and lead to bowel function and pain.
Fond et al. [10], Moser et al. [11]	Meta-analysis	Studies have shown that 60% of IBS patients have major psychosocial problems, although the specific etiology remains inconclusive. Psychosocial factors influence physiological factors such as motor function, sensory perception, and stress reactivity. Much evidence has also shown the relationship between IBS, stress, anxiety, and depression. The gut microbiota has a link with the bidirectional interaction between the gut and the nervous system. Various meta-analyses have been done to show the efficacy of psychological treatment in IBS.
Saha et al. [5], Crowell et al. [12]	Review	There is a lot of evidence that proves serotonin (5-HT), modulated via 5-HT3 and 5-HT4 receptors, plays a significant role in the control of gastrointestinal secretion, sensation, perception of visceral stimulation at peripheral and central locations, and motility. Dysfunction in these receptors can lead to intestinal and extraintestinal symptoms in IBS, such as constipation or diarrhea.
Takakura et al. [13]	Review	A large diversity of pathogenic organisms have been shown to be increased in patients with IBS, such as *Enterococcus*, *Escherichia coli*, *Klebsiella*, and *Methanobrevibacter smithii*. Gut microbiome dysbiosis causes patients with IBS to have increased permeability, dysmotility, chronic inflammation, autoimmunity, decreased absorption of bile salts, and even altered enteral and central neuronal activity resulting in abdominal pain, distension, diarrhea, and bloating.
Saito et al. [14]	Review	According to a study, relatives of someone affected with IBS has a two to three times higher risk of getting the disease. Many genetic variants have been studied in many genes, and some positive associations have been found. Estimated genetic liability is 1%-20% and heritability 0-57%.

Some well-known stressors for IBS are psychosocial factors, prior gastroenteritis, endogenous irritants, alterations in the gut microbiome, intolerance to some food, mucosal immune activation, and increased mucosal permeability [15]. There are many pharmacological and non-pharmacological treatment options for IBS, which are equally crucial for treating IBS. Tricyclic antidepressants (TCAs) are used in patients with persistent and chronic abdominal symptoms. Rifaximin is a nonabsorbable broad-spectrum antibiotic, and its use supports the idea that bacterial overgrowth is one of the pathophysiologic etiologies of IBS [16]. Pharmacological treatment is usually chosen depending on the presenting symptoms, such as mixed opiate agonists and antagonists for diarrhea, fiber and laxatives for constipation, etc.

There are studies that show non-pharmacological treatments can effectively manage IBS symptoms. Convergent standards for therapy have been proposed by some studies. Conclusions about what seems to be an effective treatment choice are based on the opinions of the specialists. A diet low in fermentable oligosaccharides, disaccharides, monosaccharides and polyols (FODMAPs) has been shown to reduce IBS symptoms in 52%-86% of patients. FODMAPs are considered to be osmotically active carbohydrates and have been theorized to contribute directly to gastrointestinal (GI) symptoms in IBS. These short-chain carbohydrates are poorly absorbed by the GI lumen and cause a variety of symptoms such as bloating, flatulence, and abdominal pain, and can increase fluid in the intestines by a direct osmotic effect [17]. Acupuncture and osteopathic medicine are examples of body-directed therapies that may be effective for overall IBS symptoms; nevertheless, higher quality randomized controlled trials (RCTs) are required to establish the therapeutic value of non-pharmacological therapy for IBS. One significant problem will be determining the best control groups to use in non-pharmacological trials.

In this review article, we will discuss in detail the importance and use of various non-pharmacological methods for treating IBS.

## Review

Role of non-pharmacological therapies in IBS 

**Figure 2:**
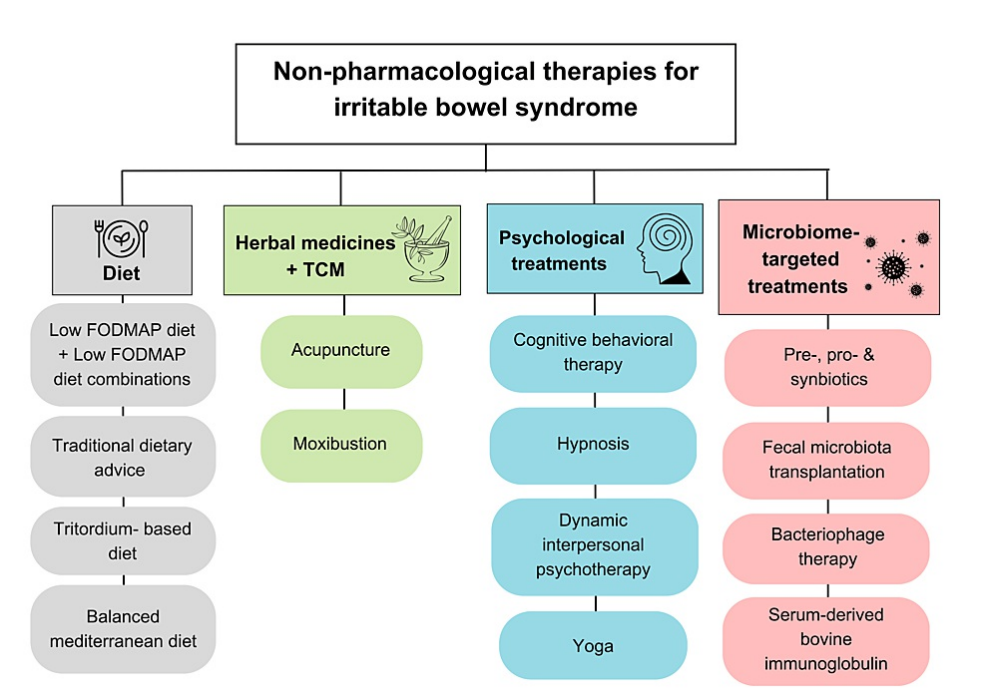
Non-pharmacological interventions for irritable bowel syndrome TCM: traditional Chinese medicine; FODMAPs: fermentable oligosaccharides, disaccharides, monosaccharides, and polyols Original figure created by Maleesha Jayasinghe

A list of the non-pharmacologic interventions that can be used in the management of IBS is depicted in Figure 2.

Dietary interventions

Diet plays a pivotal role in the pathophysiology of IBS. The effects of diet appear to be a result of an interaction between the gut bacteria and the gut endocrine cells [18]. In the small and large intestines of patients with IBS, the cell densities of Musashi 1 (a marker for intestinal stem cells and their early progeny) and neurogenin 3 (a marker for early intestinal endocrine cell progenitors originating from stem cells, which are located at the base of the crypts) are lower than those of healthy subjects [18,19]. Fewer concentrations of these markers may lead to a reduction in the density of gut endocrine cells in IBS patients. It is hypothesized that this is what leads to the development of symptoms in IBS patients [18].

The choices of different food items determine the component of the intestinal bacterial flora [20]. The diet also acts as a substrate for gut bacteria fermentation, which results in several by-products or metabolites. These by-products decrease the gut stem cells, and consequently, endocrine cell numbers, which may result in IBS [18].

Low-FODMAP Diet

FODMAPs refer to carbohydrates that are incompletely digested or absorbed in the small intestine. When they reach the colon, they are rapidly fermented by gut bacteria, producing gases. They are believed to have a potent osmotic impact by raising the water content in the small intestine and colon, causing gut distension, which may be the cause of the development of abdominal pain in IBS patients [21]. Short-chain fermentable carbohydrates (FODMAPs) were found to cause symptoms in IBS patients in a double-blind, randomized, quadruple-arm, placebo-controlled rechallenge study involving 25 patients. In contrast to 14% of patients getting glucose, 70% of patients receiving fructose, 77% of patients receiving fructans, and 79% of patients receiving a combination reported that their symptoms were not adequately controlled [22]. Additionally, they were adduced to changes in the metabolome. FODMAPs change histamine levels and the microbiota in some patient subgroups, which may affect symptoms. In a study by McIntosh et al., IBS patients were randomized to either a low- or high-FODMAP diet for three weeks, during which time their symptoms and metabolome were assessed. As opposed to the high-FODMAP group, the IBS symptom severity score (SSS) was reduced in the low-FODMAP diet (LFD) group. When compared to the high-FODMAP group, lactulose breath tests (LBT) revealed a slight reduction in H2 production in the low-FODMAP group. The low-FODMAP group had an eightfold decrease in histamine (a marker of immune activation) levels. In contrast to high-FODMAP diets, which reduced the relative abundance of bacteria implicated in gas production, low-FODMAP diets increased the richness and diversity of Actinobacteria [23].

With the exception of probiotics, the volume of evidence for the mechanisms and clinical effectiveness of the low-FODMAP diet has grown significantly over the past 10 years. Multiple fermentable oligosaccharides (fructans, galacto-oligosaccharides, or GOS), monosaccharides (fructose when in excess of glucose), disaccharides (lactose), and polyols (e.g. sorbitol, mannitol) are restricted in the low-FODMAP diet [24]. However, a strict low-FODMAP diet should only be followed for the first four to six weeks as research indicates that following a strict low-FODMAP diet for an extended period of time may have a negative effect on the intestinal microbiome. Following the initial strict period, a dietitian should be consulted to achieve the overall objective of a relaxed FODMAP restriction that allows for the addition of prebiotic FODMAPs while still maintaining symptom relief [25].

A low-FODMAP diet successfully reduced functional gastrointestinal symptoms in IBS patients; according to Halmos et al., for one week, dietary data were collected from 30 IBS patients and eight healthy people. The subjects were then randomly allocated to groups that received either a low-FODMAP diet or a typical Australian diet for 21 days, followed by a washout period of at least 21 days before switching to the alternate diet. A visual analog scale was used to evaluate daily symptoms. Subjects with IBS who consumed a low-FODMAP diet had lower overall gastrointestinal symptom scores than those who consumed a typical Australian diet or the subjects' usual diet. Bloating, pain, and wind passage were also decreased in the low-FODMAP diet group [26].

In another study, 180 Chinese patients with IBS-D were randomly assigned to an LFD group or a traditional dietary advice (TDA) group. The study's primary endpoint was a ≥50-point reduction in the IBS-SSS at three weeks. Changes in short-chain fatty acids (SCFAs) and microbiota profiles were examined in fecal samples taken before and after the dietary intervention. The primary endpoint was met in a similar number of LFD and TDA patients. Patients in the LFD group, however, experienced symptomatic relief in stool frequency and excessive wind more quickly than those receiving TDA. LFD decreased carbohydrate-fermenting bacteria like *Bifidobacterium* and *Bacteroides*, as well as saccharolytic fermentation activity, both of which were related to symptom improvement [27].

Children with IBS-related gastrointestinal symptoms have been shown to benefit from a reduced FODMAP diet. The baseline microbiome composition of those who responded significantly to the diet appears to be different from that of those who did not, with a higher saccharolytic capacity. Future studies may clarify whether gut microbiome analysis can help IBS patients receive individualized reduced FODMAP or other dietary intervention treatment [28].

A low-FODMAP diet not only reduces the symptoms of IBS but also improves the health-related quality of life (QoL) of IBS patients. A diet low in FODMAPs compared to a modified diet suggested by the National Institute for Health and Care Excellence (mNICE) led to significantly greater improvements in health-related QoL (15.0 vs. 5.0; 95% CI, -17.4 to -4.3), anxiety (95% CI, 0.46-2.80), and activity impairment (-22.9 vs. -9.44; 95% CI, 2.72-24.20) in individuals with IBS-D at four weeks [29].

Low-FODMAP Plus Gluten-Free Diet

It has been demonstrated that reduced FODMAP diet and gluten-free diet (GFD) together can lower anxiety and enhance the quality of life in IBS patients. In a recent study, 50 IBS patients were instructed to adhere to a low-FODMAP, strict GFD for six weeks. They were then randomly allocated to one of three groups A, B, or C for another six weeks. For two weeks, subjects in group A received 8 g/day of gluten; those who could tolerate gluten were given 16 g/day and then 32 g/day for a further two weeks; group B followed a low-FODMAP, strict GFD; and group C received a diet containing gluten. After the first six weeks, the satiety score in group C considerably rose while the GFD group's pain severity, bloating, and total score significantly decreased. At the conclusion of the study, a between-group analysis showed significant differences in pain intensity, pain frequency, and impact on community function [30].

Another study found that individuals who consumed an LF-GFD experienced a substantial reduction in the severity of their IBS symptoms and a normalization of their gut microbiota (GM). Forty-two IBS patients who met the Rome IV criteria received LF-GFD treatment for six weeks. In comparison to the baseline, the IBS-SSS was considerably lower after the LF-GFD intervention. In fecal samples, significant microbe variations between the intervention's first and second phases were found. Following the dietary intervention, there was a significant increase in Bacteroidetes and a large decline in the Firmicutes to Bacteroidetes (F/B) ratio. After a six-week dietary intervention, the fecal calprotectin levels significantly dropped [31].

More clinical trials are required to prove the long-term efficacy and safety of LF and GFD dietary combinations in order to create personalized diet plans for individuals with IBS. The benefit of GFD in IBS may actually be from fructan restriction rather than from gluten itself; therefore, more studies are needed to investigate this.

Low-FODMAP With Bacillus coagulans Supplement Diet

In a study by Abhari et al., 50 IBS patients who fulfilled Rome IV criteria were randomized to an eight-week low-FODMAP diet along with either a probiotic or a placebo capsule. Both groups experienced significant improvements in the quality of life, consistency of defecation, frequency, and intensity of abdominal pain, and patient-reported severity score. However, only the improvement in severity scores was noticeably greater in the probiotic group when compared to the placebo group. In addition, compared to the placebo group, the probiotic group had a substantially higher frequency of patients who experienced a clinical improvement in IBS-SSS [32].

Traditional Dietary Advice, Tritordeum-Based Diet, and Balanced Mediterranean Diet

TDA, LFD, and GFD are effective approaches in non-constipated IBS. The TDA diet places a focus on regular eating habits with a lowered intake of fatty and spicy meals, caffeine, alcohol, and other foods that frequently cause symptoms [28]. A study conducted by Rej et al. found that TDA is the most patient-friendly in terms of cost and convenience. Therefore, the study recommends TDA as the first-choice dietary therapy in non-constipated IBS, with LFD and GFD reserved according to specific patient preferences and specialist dietetic input. In this study, the primary endpoint of a ≥50-point reduction in IBS-SSS was met by 42% undertaking TDA, 55% for LFD, and 58% for GFD [33].

In another study when an LFD and a TDA were given to 108 IBS-D patients (Rome III criteria), each type of diet reduced symptoms in IBS-D patients; however, the LFD achieved earlier symptomatic improvements in stool frequency and excessive wind. The therapeutic effect of the LFD was associated with changes in the fecal microbiota and the fecal fermentation index. At baseline, the presence of severe symptoms and microbial metabolic dysbiosis characterized by high saccharolytic capability predicted favorable outcomes of the LFD intervention [27].

Tritordeum-based diet may represent a valid alternative, with high palatability, especially among Italian patients, for whom pasta is considered one of the main assets of dietetic culture, and would be easier to manage in their daily habits. In a randomized controlled trial that compared the effects of 12 weeks of LFD to TBD in improving the symptom profile of IBS-D patients, the two diets equally improved gastrointestinal symptoms and QoL, measured by the IBS Severity Scoring System questionnaire, reducing the total score after four weeks and maintaining this range until the end of treatment. Therefore, TBD has a place in the management of IBS, especially in Italian patients [34].

The Mediterranean diet (MD) is also a safe dietary option that can be used to manage symptoms of IBS. It is characterized by a low content of saturated fatty acids, high monounsaturated fatty acids, high amounts of fiber, complex carbohydrates, and essential antioxidants. MD was associated with a significant improvement in IBS scores and quality of life in children and adolescent patients with IBS in a prospective, cross-sectional case-controlled study that included 100 IBS patients [35]. However, further clinical trials are required to identify the role of MD in IBS.

Microbiome-targeted therapies

Recent research has explored the possibility of altering the gut microbiota by diet and using probiotics, prebiotics, and synbiotics to treat IBS with encouraging findings [36].

Prebiotics

Prebiotics are defined as fibers that stimulate the growth of beneficial gut microflora and are resistant to enzymatic digestion in the human gastrointestinal tract (GIT) [8]. The mechanisms of action of prebiotics are aimed at reducing inflammation through the formation of short-chain fatty acids [37]. These can be obtained from various natural sources such as cereals, fruits, green vegetables, and plants. Some are synthetically produced and these include lactulose, malto-oligosaccharides, galacto-oligosaccharides, fructo-oligosaccharides (FOS), lactosaccharose, cyclodextrins [36]. At present, research is being conducted regarding the effectiveness of various new classes of prebiotics for the treatment of IBS including resistant starch, arabinoxylan-oligosaccharides, manno-oligosaccharides and xylo-oligosaccharides [38]. Research has shown that utilization of fungal metabolites such as beta-glucans shows promise in the treatment of IBS patients. It has been found to decrease symptoms of abdominal pain and bloating and also alleviate visceral pain along with a reduction of restraint stress-induced fecal pellet output [39].

According to a study conducted by Silk et al., investigating the effects of a prebiotic mixture of trans-galacto-oligosaccharide (TGOS) on patients with IBS, usage of TGOS showed significant improvements in stool consistency, flatulence, bloating, composite symptom score, and subjective global assessment compared to the baseline [40]. The reason for this phenomenon is that pre-biotic galacto-oligosaccharides have the ability to undergo targeted digestion by *Bifidobacteria*, thereby facilitating the proliferation of *Bacteroides*, *Lactobacilli*, and particularly, *Bifidobacterium* [41].

On the other hand, Wilson et al. conducted a systematic review to assess the impact of prebiotics on global response, gastrointestinal symptoms, quality of life, and gut microbiota in adult patients with IBS and other functional bowel disorders. According to this study, the group of patients who were administered prebiotics did not exhibit any significant variations in the intensity of abdominal pain, bloating, flatulence, and overall quality of life in comparison to the placebo group [42].

Furthermore, it is to be noted that the administration of prebiotics is not advised for the management of functional gastrointestinal disorders (FGIDs) in pediatric patients. A study conducted in a sample of 71 children by Levy et al., which administrated a prebiotic treatment consisting of 900 mg inulin twice daily for a duration of four weeks, did not yield significant results [43].

The dose of the prebiotic used is a crucial factor in the treatment of IBS. Based on data obtained from four clinical trials conducted on adults, it has been observed that lower doses may be effective and high doses may have a negative impact on symptoms [43,44].

Limited data exist on the efficacy of prebiotics in the context of IBS, despite the extensive research conducted. Evidence from further trials are required to establish the effectiveness and safety of these treatments in managing IBS [37].

Probiotics

A United Nations and World Health Organization Expert Panel defines probiotics as “live microorganisms which when administered in adequate amounts confer a health benefit on the host” [45]. By producing antimicrobial substances and hindering their attachment to the mucosa of the gut, they are seen to decrease the growth of pathogens [36]. According to research, certain probiotic bacterial genera such as *Lactobacillus* and *Bifidobacterium* have been found to reduce colonic hypersensitivity by increasing the expression of μ-opioid and cannabinoid receptors [8]. A further intriguing idea in the world of novel probiotics is the addition of "archeabiotics" or soil-based probiotics for FGIDs [36].

A systematic review conducted by Dale et al. identified seven RCTs (63.6%) that showed significant improvement in patient groups in which probiotics were used compared to the placebo [46]. Ford et al. conducted a systematic review that indicated that certain probiotic combinations, or specific species and strains such as *Lactobacillus plantarum* DSM 9843, *Escherichia coli* DSM 17252, and *Streptococcus faecium* appeared to have favorable outcomes in abdominal discomfort and overall IBS symptoms [47]. The observed effect of probiotics is commonly attributed to the specific strain of microorganisms utilized. Hence, it is not possible to generalize the impact of a particular probiotic to another probiotic belonging to the same species or even to a distinct strain. Furthermore, the effects of probiotics may vary across different demographic groups, as well as in relation to the progression and categorization of illnesses [48]. It was also found that formulations containing multiple strains of species might be more beneficial compared to those with single strains [49]. Nevertheless, the comprehension of the precise mechanisms of probiotic microorganisms remains limited [50].

Synbiotics

Synbiotics are a combination of probiotics and prebiotics that work symbiotically to improve the health of the host, when introduced to the gut it should specifically stimulate the growth and metabolism of a physiologic microbiota in the intestine that has a beneficial impact on IBS patients [36,38]. The most popular probiotic strains (*Lactobacillus* and *Bifidobacterium*) make up the majority of the synbiotics [49]. Inulin with *Bifidobacterium lactis*, *Saccharomyces boulardii* with ispaghula husk, and yogurt with acacia fiber and *B. lactis* are a few examples of synbiotics utilized in IBS that have positive results [20]. In a study assessing the effectiveness of the synbiotic combining *Lactobacillus paracasei* DKGF1 and *Opuntia humifusa* in 78 elderly individuals with IBS, it was found that the synbiotic greatly reduced the symptoms of IBS, such as bloating, stomach pain, and irregular stools. The synbiotic was well-tolerated by the patients and had no negative effects. These results are in line with a growing body of research that shows how effective probiotics and synbiotics are at treating IBS. The study's only drawback is that it only covered senior individuals, so it is possible that the findings cannot be applied to people of different ages. To assess the ideal course of treatment and the possible advantages of long-term use, more research is required [51].

Lee et al. investigated how IBS patients responded to a different synbiotic product that contained probiotic bacteria from the *Lactobacillus* and *Bifidobacterium* genera, FOS, and inulin. The study team noticed reductions in trial participants' bloating, exhaustion, and abdominal discomfort [52].

More data are required to support the advantages of synbiotics in the management of IBS because there have only been a few clinical trials conducted so far [38].

Fecal Microbiota Transplantation

The fecal microbiota transplantation (FMT) procedure entails the infusion of fecal microbiota material from a healthy donor into the gut of a recipient patient as a form of treatment [53]. As there is evidence indicating that abnormal gut microflora plays a pivotal role in the development of IBS, the approach provided by FMT presents an innovative method for the restoration of gut microbiota in individuals affected by IBS [54].

The precise mechanism of action of FMT still remains unclear. However, it is postulated that FMT seeks to re-establish the normal balance of immunological and inflammatory responses, neurotransmitters and vasoactive chemicals, and energy metabolism, with the ultimate goal of restoring the intestinal flora [55]. The success of FMT is affected by the attributes of the donors, leading to the inference that the mere presence of microbial diversity cannot be deemed a dependable predictor of favorable outcomes [56]. Furthermore, the effectiveness of FMT treatment can be influenced by external factors, such as dietary behaviors, which may contribute to variable outcomes. The avoidance of fibrous foods by patients may exert a substantial influence on the metabolism and functionality of recently acquired microbiota [57]. The effectiveness of FMT in treating IBS was found to be dependent upon the precise identification of suitable donors and the selection of appropriate routes for fecal administration, as per the existing literature [58]. A sub-group analysis in a systematic review conducted by Rodrigues et al., which included 489 adult IBS patients, showed that the effectiveness of FMT delivered through colonoscopy, gastroscopy, and nasojejunal tube is significantly influenced by the subtype of IBS [2].

A meta-analysis found that FMT resulted in a significant improvement in IBS-SSS and IBS-QoL in patients diagnosed with IBS in the short term [58]. Another study conducted by Goll et al. including 14 participants from a previously conducted REFIT trial concluded that long-term changes in the gut micro-flora were seen when FMT was used, and hence supported the use as a potential therapeutic option for IBS [59].

However, a meta-analysis conducted by Wu et al., involving 472 patients with IBS, revealed that FMT did not result in a significant improvement in global IBS symptoms at 12 weeks when compared to a placebo. Furthermore, the results of subgroup analyses indicated that the efficacy of FMT was greater than that of placebo when delivered through colonoscopy or gastroscopy. It is worth noting that, when FMT was administered through oral capsules, it was found to be less effective than the placebo [60].

The optimal dosage and frequency of FMT for individuals suffering from IBS remain undetermined [61]. Future research is warranted to determine the appropriate selection criteria for IBS patients who are suitable candidates for FMT, as well as to identify the optimal donor microbiota for achieving therapeutic efficacy. It is also imperative to establish the most effective FMT protocol for IBS. If antibiotic pretreatment is required or not and the required frequency in which FMT should be repeated has not been conclusively demonstrated [53]. In general, there have been no reported instances of severe adverse events associated with FMT [60]. The evaluation of the safety of FMT is currently in its early phases, lacking a broad consensus and necessitating further investigation [57].

Bacteriophage Therapy

Galtier et al. conducted a study on a mouse model and reported that a combination of phages effectively alleviated colitis symptoms in patients with Crohn's disease [62]. However, there exists a lack of established data regarding the use of viral treatment of IBS. A study examining latent herpes virus infection revealed that viruses have the ability to modulate the immune status for the betterment of individuals with IBS [39]. The utilization of these agents for therapeutic purposes presents numerous benefits, including the notable specificity of bacterial taxa, the co-adaptation of bacteria resulting in reduced resistance, and the cost-effective and straightforward production process. Nevertheless, there exist significant limitations, primarily pertaining to legal and ethical concerns associated with the potential for inducing septic/toxic shock [63].

Hence, it is imperative to conduct longitudinal metagenomic investigations and clinical trials to evaluate their prospective advantages and drawbacks. Enhancing comprehension and application of the interplay between intestinal microbiota holds significance in reestablishing intestinal equilibrium in individuals with IBS. This includes the dissolution of pathogens and transmission of antipathogenic characteristics to commensal bacteria by specific viruses [64].

Serum-Derived Bovine Immunoglobin

The medicinal food, serum-derived bovine immunoglobulin (SBI), which is a mixture constituting IgG, has been granted approval by the US Food and Drug Administration for the treatment of patients with IBS-D [65]. EnteraGam®, which contains SBI, is a prescription-based medical food that is administered under close supervision. It is designed to treat disorders of the intestine in patients who experience chronic recurrent episodes of passing loose stools, and who have a restricted or compromised capacity to consume, digest, absorb, or assimilate certain nutrients [66].

SBI is believed to work by involving the participation of zonula occludens-1 (ZO1) and occludin, which are tight junction proteins. Moreover, they attach to components of gut microbes, regulate immune homeostasis in the gastrointestinal tract, control intestinal barrier function, and improve nutrient absorption [67]. Valentin et al. conducted a study that revealed that SBI therapy was connected with many alterations in the abundance of Proteobacteria Burkholderiales and Firmicutes Catonella in samples obtained from brush biopsy of the duodenum, but no modifications in the microbiota of the stool were to be found. The aforementioned discoveries suggest that the utilization of SBI medication could potentially impact the microbiome of the small intestinal mucosa [68]. The utilization of SBI in IBS-D is supported solely by limited evidence such as case reports, along with a clinical trial that was limited in power [65]. More research needs to be conducted in this regard before it can be established as a standard therapeutic option.

Alternative and complementary medicine therapies

No specific test can identify IBS; thus, no one can know whether they have it, as experts have yet to determine its etiology. Hence, the treatment options vary according to geography and medicinal practices worldwide. A study used network pharmacology technology to build a system and anticipate how components of Shenling Baizhu powder (SLBZP), a type of traditional Chinese medicine (TCM), would interact with target genes. SLBZP might have a role in both the processes that treat IBS and those that prevent it from arising. The findings suggest that SLBZP may effectively treat IBS by modulating the tumor necrosis factor (TNF) signaling system [69].

In TCM, the Tongxie formula is the most commonly prescribed medication for IBS. To conclude the use of the post-treatment therapeutic effects (PTTEs) of Tongxie in IBS therapy, the study gathered outcome data during the Tongxie therapy (which lasted four weeks) and after it (for 57 weeks) in order to appropriately quantify PTTEs. Tongxie quickly relieved patients' IBS symptoms, with stool consistency falling by 73.0% on day 3, pain scale declining by 75.8%, and secondary endpoints falling to 73.2% (pain frequency) and 78.1% (stool frequency). The study found that the proportion of patients with abdominal pain was reduced, and the percentage of patients with improved stools remained stable. Tongxie could be a successful alternative therapy for IBS patients who do not respond well to traditional therapies, potentially shedding light on effective intermittent IBS treatment [70].

Only a few studies have specifically studied the usefulness of acupuncture (ACU) and pinaverium bromide in treating IBS together. According to these studies, only about one-third of American IBS sufferers are happy with how their therapy is working out. Since their symptoms did not dramatically improve, several patients also stopped taking the drug because of dissatisfaction. Patients have turned to ACU with drug combinations [71]. Although herbs and spices have long been used to treat gastrointestinal and painful conditions, it is unknown whether they are useful in the treatment of functional abdominal pain disorders (FAPDs), particularly in young people. Large, randomized, placebo-controlled trials are required to examine the safety and efficacy of herbs and spices in the treatment of FAPDs. This can broaden the therapeutic options and provide specific therapies for those suffering from functional gastrointestinal pain symptoms [72].

The latest development in Chinese herbal medicine (CHM) for IBS can effectively treat IBS symptoms and stomach pain, but it is more likely than a placebo to cause negative effects. However, most adverse reactions were modest and did not necessitate further medical attention. Randomized controlled trials of CHM for IBS suggest that CHM could be a relieving therapy for those with IBS [73]. According to the latest development in the efficacy and safety of a novel herbal medicine in the treatment of IBS, the herbal drug under consideration was more effective than hyoscine at alleviating IBS symptoms. As a result, it can be regarded as an alternative kind of IBS treatment [74]. After a complete meta-analysis of the impact of peppermint oil on IBS treatments, enteric-coated peppermint oil (PO) was found to be a safe and effective treatment for abdominal pain and overall generalized symptoms in adults with IBS. Powerful findings were shown by the large effect size of PO over placebo in the alleviation of abdominal pain and overall symptoms after analyzing the largest cohort of RCTs published over five decades, encompassing 12 randomized clinical trials with 835 IBS patients from all over the world [75].

The first placebo-controlled experiment to look at the effect of low-adsorbable geraniol on IBS symptoms and gut microbiota suggests how the symptoms are effectively improved. According to the study's major finding, geraniol significantly reduced overall IBS symptoms and improved the GM profile. The researchers used the irritable bowel syndrome symptom severity score, which is a composite instrument, to assess clinical efficacy [76].

According to the latest traditional Chinese medicine interference, the findings of a small randomized clinical trial on acupuncture treatment for IBS-D showed a clinically significant reduction in IBS-D symptoms in both specific acupoints (SA) and non-specific acupoints (NSA) and non-acupoints (NA) with a 1:1:1 ratio, suggesting acupuncture is a viable and safe therapy option for IBS-D [77]. However, more considerable research with sufficient power is necessary to evaluate acupuncture's efficacy for IBS-D accurately. The study also proposed that the FDA-recommended composite response rate can be used as the primary outcome indicator for the trial. This study suggested that ACU may help treat IBS-D, but more research is needed to confirm this initiative. Acupuncture for IBS effectively lowers visceral hypersensitivity and alters the gut-brain axis. On the other hand, 16 clinical trials in IBS showed a significant placebo response rate [77]. Acupuncture has been used by patients seeking relief from the side effects of medication and traditional IBS therapy and has been found to be effective in the treatment of GIT illnesses [71].

Moxibustion (MOX), ACU combination therapy, and electroacupuncture (EA) may be the most effective in lowering anxiety and sadness in IBS-D patients, among the several interventions studied. Combining ACU-related therapies with other treatments appears to have a high overall benefit and is considered safe [78]. The selection of acupoints in TCM influences how well acupuncture treatments work. Bilateral points located in the upper abdomen 2 cun (cun is the TCM unit of length, measured as the width of a person's thumb at the knuckle) lateral to the center of the umbilicus (Tianshu, ST25), located below the knee, on the tibialis anterior muscle (Zusanli, ST36), and on the lower leg, one handbreadth below the tibial tuberosity and one finger's width lateral to the anterior crest of the tibia (Shangjuxu, ST37) are crucial for reducing diarrheal symptoms [78]. In contrast, acupuncture therapy for psychosomatic diseases focuses on specific acupoint functions. An acupoint on the anterior forearm, 2 cuns above the wrist crease, between the tendons of the palmaris longus and flexor carpi radialis muscles (Neiguan, PC6) is used in the treatment in addition to ST25, ST36, and ST37 because clinical studies demonstrate that it helps relieve anxiety symptoms [79].

Acupuncture is complementary and alternative medicine that is widely used as an adjuvant therapy to reduce adverse drug effects and improve therapeutic outcomes. Studies have indicated that acupuncture can help with IBS symptoms [80]. Acupuncture and/or moxibustion have been shown to help patients with IBS by reducing the severity of symptoms, relieving stomach pain, and improving quality of life. In sham control trials, acupuncture did not yield strong, conclusive evidence, but moxibustion produced effects with substantial heterogeneity. More rigorous sham control trials, the authors warn, are needed to evaluate if acupuncture or moxibustion is beneficial in treating IBS [81,82].

Combining septum moxibustion with acupuncture improves the quality of life and increases the total effective rate. However, the dependability of combining septum moxibustion with acupuncture is low due to inadequate methodological and randomized controlled trials. Future research must have a stricter design and uniform reports. More research and meta-analyses are needed to assess how effective acupuncture is in treating IBS [82].

Psychological therapies

Skype hypnotherapy, like traditional hypnosis, was found to improve all of the particular IBS symptoms as well as noncolonic symptoms, quality of life, anxiety, and depression scores. Many drugs, on the other hand, simply treat one or two symptoms, such as pain or gastrointestinal distress. According to the FDA criteria, abdominal discomfort is the most common IBS symptom that needs to be treated [83].

In a study by Lackner et al., in contrast to individuals who got IBS education (EDU), patients who received cognitive behavior therapy requiring minimal therapist contact (MC-CBT) had a significant improvement in gastrointestinal symptoms two weeks after treatment. A total of 61% of patients and 55.7% of gastroenterologists who gave the treatment a favorable review rated the improvement as moderate to considerable. Patients who received EDU, on the other hand, saw a slight improvement in symptoms, with 43.5% of patients and 40.4% of gastroenterologists assessing the change favorably. Six months after therapy, gastroenterologists assessed the improvement in gastrointestinal symptoms and discovered a significant difference in improvement between the MC-CBT group and the EDU group [84].

A study used a theoretical framework to investigate how gastroenterologists and patients perceived yoga as a treatment for IBS; Theory of Planned Behavior (TPB) variables were used to explain IBS patients' attitudes toward yoga. Despite having positive attitudes and notions about yoga, gastroenterologists' prescription patterns did not reflect these attitudes and beliefs, showing a gap between their thoughts and actions [85].

We already know that there are several systemic explanations for IBS symptoms and that mental health disorders can aggravate or exacerbate these causes. To evaluate if psychological counseling techniques may be used to alleviate IBS symptoms. A variety of psychiatric therapies have been reported to be beneficial in treating IBS, although none have been found to be more effective than others. Among these treatments, CBT-based interventions and gut-directed hypnotherapy had the greatest evidence to support them and were the most beneficial in the long run [86]. A randomized controlled experiment found that a home-based variation of CBT was more effective than education at dramatically and persistently reducing gastrointestinal symptoms in IBS patients [84]. Treatment options for IBS are undetermined. However, new research trials on TCM reveal that TCM has a vital function in modulating gastrointestinal motility and its pathogenesis. This discussion aims to give insights into "how new inventions over the last few years' fluctuations affected the effectiveness of IBS treatment."

Limitations

This analysis is based on a review of research papers published between 2004 and 2023; hence, we may have overlooked important information from research articles published prior to 2004. Furthermore, because our analysis only included papers written in English, we may have missed studies published in other languages.

## Conclusions

IBS is thought to affect 5%-10% of people worldwide, and the majority of IBS patients are under the age of 50. The precise cause of IBS is unclear. Symptoms may be caused by a disruption in the interaction of the gut, brain, and neurological systems. Changes in typical bowel movements and sensations may result from this. IBS treatments are available to help manage symptoms. However, not all treatments are effective for everyone. This study provides an overview of the non-pharmacological treatments that can be utilized to control IBS.
